# Coordination complexes of zinc and manganese based on pyridine-2,5-dicarboxylic acid *N*-oxide: DFT studies and antiproliferative activities consideration†

**DOI:** 10.1039/d1ra08258b

**Published:** 2021-11-22

**Authors:** Hanie Alizadeh, Masoud Mirzaei, Amir Sh. Saljooghi, Vida Jodaian, Maryam Bazargan, Joel T. Mague, Rosa M. Gomila, Antonio Frontera

**Affiliations:** Department of Chemistry, Faculty of Science, Ferdowsi University of Mashhad Mashhad 9177948974 Iran mirzaeesh@um.ac.ir; Department of Chemistry, Islamshahr Branch, Islamic Azad University Islamshahr 3317843154 Iran; Department of Chemistry, Tulane University New Orleans LA 70118 USA; Departament de Química, Universitat de les Illes Balears Crta de Valldemossa km 7.5 07122 Palma de Mallorca (Baleares) SPAIN

## Abstract

We report here the design, synthesis, and antiproliferative activity of three coordination complexes [Mn_2_(pydco)_2_(bpy)_2_(H_2_O)_2_]·2H_2_O (1), [Zn(bpy)(Hpydco)_2_] (2), and [Zn(bpy)Cl(Hpydco)]·2H_2_O (3) (H_2_pydco = pyridine-2,5-dicarboxylic acid *N*-oxide, bpy = 2,2′-bipyridine). Molecular structures of these complexes have been characterized by elemental analysis, Fourier transform infrared spectroscopy, thermogravimetric analysis, and powder and single-crystal X-ray diffraction. According to the structural analysis, 1–3 are discrete complexes containing *N*- and *O*-donor ligands (bpy and pydco^2−^) in which pydco^2−^ can be coordinated to the metal centres *via* the *N*-oxide oxygen and one carboxylate oxygen to generate a six-membered chelate ring. Also, these structures benefit from extensive intermolecular interactions such as hydrogen bonds and π-interactions which are the major forces to make them more stable in the solid state. The energetic features of the π-stacking interactions observed in compounds 1–3 have been computed and compared to the H-bonds. The interactions in the solid state have been also studied using the independent gradient model approach (IGM plot). The IGM-δg approach uses a new descriptor (δg) that locally represents the difference between a virtual upper limit of the electron density gradient and the true electron density gradient. This newly developed IGM methodology automatically extracts the signature of interactions between two given fragments. Finally, the antiproliferative properties of these complexes were tested on several cancer cell lines by MTT assay and flow cytometry. Also, to compare the antiproliferative activities of these complexes with common chemotherapy drugs, the antiproliferative property of cisplatin was evaluated as a reference and positive control.

## Abstract 2

In this manuscript we analysed the coordination complexes of zinc and manganese with pyridine-2,5-dicarboxylic acid *N*-oxide, focusing on the supramolecular assemblies and antiproliferative activities

## Graphical abstract

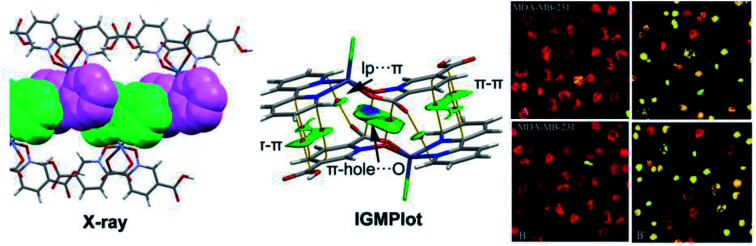

## Introduction

Nowadays, metal-based drugs are known as an important class of anti-tumor agents. They can be classified into two groups; (i) those containing the classical noble metals such as Pt, Ru, Au, Ti, and Pd which are the most promising candidates in the treatment of several tumors and (ii) those of low-cost, bio-essential metals such as Cu, Fe, Zn and Mn.^1–8^ The former group suffers from some clinical problems such as toxicity, solubility and drug half-life while the latter group are better dealt by human physiology and cause less damaging side effects. Despite the variety of physiological roles of the zinc and manganese ions and their complexes, they have been less studied as metal-based anticancer drugs.

Apart from the choice of the metal ion, the structure of the organic ligand(s) can have a great influence on the final biological properties by limiting the adverse effects of metal overload, inhibiting selected metalloenzymes and facilitating metal ion redistribution. Up to now, a variety of ligands with various donor atoms have been introduced for bio-essential metals. Among them, *O*/*N*-donor ligands are almost the most representative category. Many strategies such as utilizing of one- or mixed-ligand complexes have been reported and due to the variety of accessible arrangements, different probable frameworks have been employed as anticancer agents. For the metal complex-based drugs, two groups of organic ligands are primarily employed: (i) aromatic bidentate *N*-donors such as 2,2′-bipyridine and 1,10-phenanthroline which are used in molecular biology as DNA intercalation agents, as contrast agents for magnetic resonance imaging, chelation therapies, antiproliferative,^9,10^ and metalloenzymes. (ii) pyridine polycarboxylates with *N*/*O* donor properties which have vast and remarkable applications such as anticancer properties, anti-HIV agents, gas adsorbents, magnetism and luminescence to name just a few.^11–16^

Recently, the chemistry of pyridine-2,5-dicarboxylic acid (H_2_pydc) has attracted attention,^17–21^ due to the position of its carboxylates and aromatic rings which is not only interesting for its diverse coordination complexes but also its potential to create supramolecular structures through intermolecular interactions such as π-stacking and hydrogen bond.^12,22,23^ It is important to note that *N*-oxidation of the pyridine ring in the H_2_pydc can produce an *O*-donor ligand (pyridine-2,5-dicarboxylic acid *N*-oxide (H_2_pydco)) with better electron donor affinity than the nitrogen atom of the pyridine ring in H_2_pydc because the neutral nitrogen atom can donate one pair of electrons, while the charge-polarized pyridine-*N*-oxide moiety can donate three pairs of electrons.^24^ According to the literature survey, this feature can enhance the CO_2_ separation properties of isoreticular MOFs.^11^ Several reviews reflect the huge potential of metal-based frameworks as drug delivery and cancer theragnostic platforms.^25–28^

As part of our study on bio-essential metal complexes as antiproliferative agents, we have synthesized three coordination complexes with H_2_pydco and bpy under similar condition which are formulated as [Mn_2_(pydco)_2_(bpy)_2_(H_2_O)_2_]·2H_2_O (1), [Zn(bpy)(Hpydco)_2_] (2), and [Zn(bpy)Cl(Hpydco)]·2H_2_O (3). The structural description of the three compounds has been done and the interesting π-stacking assemblies have been studied using DFT calculations. In particular, the energetic features of several assemblies have been computed and compared. The interactions in the solid state have been also characterized using the independent gradient model approach (IGM plot), which is a novel methodology to reveal noncovalent interactions in real space. Finally, the antiproliferative potential of 1–3, have investigated against MDA-MD-231, MCF-7, HeLa, HT-29, Neuro-2a cancer cell lines also L-929 as a normal cell, using MTT and TUNEL assay and flow cytometry test.

## Experimental

### Materials and instruments

All chemicals and solvents were purchased from commercial sources and used without further purification, except for pyridine-2,5-dicarboxylic acid *N*-oxide (H_2_pydco) which was synthesized according to a reported method.^29^ Melting points were determined using a Barnstead Electrothermal 9300 apparatus. The infrared spectra were recorded in the range of 4000 to 400 cm^−1^ on a Thermo Nicolet/AVATAR 370 Fourier transform spectrophotometer using KBr pellets. Elemental analysis (CHN) was performed using a Thermo Finnigan Flash-1112 EA microanalyzer. Metal content was measured by the Spectro Arcos ICP-OES spectrometer model 76004555 using in the range of 130–770 nm for ICP spectra. Thermal gravimetric analysis (TGA) was carried out under an air atmosphere from ambient temperature up to 950 °C with a heating rate of 10 °C min^−1^ on a Shimadzu TGA-50 instrument.

### Synthesis and characterization

#### Synthesis of 1

A solution of bpy (0.031 g, 0.2 mmol) in methanol (6 ml) was added to an aqueous solution of MnCl_2_ (0.025 g, 0.2 mmol) and H_2_pydco (0.03 g, 0.2 mmol) in water (6 ml) and the mixture was refluxed at 90 °C for 6 h. After 10 days, yellow crystals were obtained by slow evaporation from the reaction mixture at room temperature in 70% yield (60 mg) (based on Mn) (mp 180 °C). Anal. calcd (%) for C_34_H_30_Mn_2_N_6_O_14_: C, 47.68; H, 3.53; N, 9.81; Mn, 12.61. Found (%): C, 47.44; H, 3.43; N, 9.72; Mn, 12.40. IR bands (KBr pellet, cm^−1^): 3481, 3082, 3019, 1715, 1639, 1594, 1473, 1438, 1390, 1315, 1224, 1154, 1053, 1014, 1014, 946, 773, 736, 648, 461, 413.

#### Synthesis of 2

Complex 2 was prepared similarly to 1, except that an aqueous solution was used instead of methanolic one and Zn(NO_3_)_2_ (0.06 g, 0.2 mmol) was used instead of MnCl_2_. Yellow crystals were obtained after 15 days by slow evaporation of the reaction mixture in 22% yield (25 mg) (based on Zn) (mp 210 °C). Anal. calcd (%) for C_24_H_16_N_4_O_10_Zn: C, 49.21; H, 2.75; N, 9.56; Zn, 11.20. Found (%): C, 49.15; H, 2.68; N, 9.46; Zn, 11.13. IR bands (KBr pellet, cm^−1^): 1329, 3082, 2782, 2627, 2500, 1717, 1650, 1597, 1507, 1395, 1290, 1210, 1155, 1123, 948, 759, 632, 609, 466.

#### Synthesis of 3

Complex 3 was prepared similarly to 1, except that ZnCl_2_ (0.027 g, 0.2 mmol) was used instead of MnCl_2_ and a 24 h reflux period was employed. After 15 days, yellow crystals were obtained by slow evaporation of the reaction mixture at room temperature in 27% yield (25 mg) (based on Zn) (mp 229 °C). Anal. calcd (%) for C_17_H_16_ClN_3_O_7_Zn: C, 42.97; H, 3.39; N, 8.84; Zn, 13.76. Found (%): C, 42.77; H, 3.28; N, 8.65, Zn, 13.70. IR bands (KBr pellet, cm^−1^): 3487, 3113, 3076, 1691, 1643, 1597, 1564, 1473, 1443, 1399, 1349, 1313, 1206, 1155, 1026, 962, 841, 765, 735, 632, 415.

### X-ray structure determination

Single crystals of 1–3 were mounted on polymer loops with a drop of heavy oil and flash cooled to 150 K in the cold nitrogen stream of an Oxford Cryostream 800 low temperature device attached to a Bruker AXS D8 Quest diffractometer equipped with a PHOTON 3 photon-counting detector. Hemispheres of intensity data were collected under control of the APEX3 (ref. 30) software with the raw data reduced to *F*^2^ values with SAINT^30^ which also performed global least squares refinements of the cell parameters. Application of numerical absorption corrections and merging of equivalent reflections was done with SADABS^31^ and the structures were solved by dual space methods (SHELXT^32^). The structures were refined by full-matrix, least-squares methods (SHELXL^33^) with hydrogen atoms included as riding contributions in idealized positions with isotropic displacement parameters tied to those of the attached atoms. For each structure, a few reflections affected by the beamstop were omitted from the final refinement. Crystal data, data collection, and refinement parameters are given in Table 1.

**Table 1:** Crystal data, data collection, and refinement parameters for 1–3

	1	2	3
Emp. formula	C_34_H_30_Mn_2_N_6_O_14_	C_24_H_16_N_4_O_10_Zn	C_17_H_16_ClN_3_O_7_Zn
Formula weight	856.52 g mol^−1^	585.78 g mol^−1^	475.15 g mol^−1^
Temperature	150(2) K	150(2) K	150(2) K
Wavelength	0.71073 Å	0.71073 Å	0.71073 Å
Crystal system	Triclinic	Monoclinic	Triclinic
Space group	*P*1̄	*P*2_1/*C*_	*P*1̄
*a* (Å)	7.1430(12)	13.7121(19)	8.5124(9)
*b* (Å)	9.3717(15)	17.737(3)	9.5030(10)
*c* (Å)	13.017(2)	9.5694(13)	12.0869(13)
*α* (°)	85.852(2)°	90	75.923(2)
*β* (°)	75.893(2)°	93.303(2)	84.467(2)
*γ* (°)	89.950(2)°	90°	80.995(2)
Volume (Å^3^)	842.8(2)	2323.5(6)	934.96(17)
*Z*	1	4	2
Density	1.688 g cm^−3^	1.675 g cm^−3^	1.688 g cm^−3^
Abs. coeff.	0.833 mm^−1^	1.127 mm^−1^	1.504 mm^−1^
*F*(000)	438	1192	484
Crystal size	0.117 × 0.121 × 0.250 mm	0.112 × 0.128 × 0.320 mm	0.196 × 0.218 × 0.250 mm
Theta range	1.62 to 27.57°	1.49 to 28.37°	1.74 to 29.32°
Index ranges	−9 ≤ *h* ≤ 9	−18 ≤ *h* ≤ 18	−11 ≤ *h* ≤ 11
	−12 ≤ *k* ≤ 12	−23 ≤ *k* ≤ 23	−13 ≤ *k* ≤ 13
	16 ≤ *l* ≤ 16	−12 ≤ *l* ≤ 12	−16 ≤ *l* ≤ 16
Reflections collected	15 261	21 604	18 159
Independent reflections	10 490	5756	5008
*R*_int_	0.037	0.0693	0.0263
Data/restraints/parameters	15 261/0/254	5756/0/352	5008/0/262
GOF on *F*^2^	1.070	1.047	1.127
Final *R* indices[*I* > 2*σ*(*I*)]	*R*_1_ = 0.0618, *wR*_2_ = 0.1660	*R*_1_ = 0.0490, w*R*_2_ = 0.1168	*R*_1_ = 0.0255, w*R*_2_ = 0.0708
*R* Indices (all data)	*R*_1_ = 0.0913, w*R*_2_ = 0.1838	*R*_1_ = 0.0798, w*R*_2_ = 0.1298	*R*_1_ = 0.0287, w*R*_2_ = 0.0717
Largest diff. peak and hole	1.139 and −0.743 eÅ^−3^	1.007 and −0.617 eÅ^−3^	0.583 and −0.284 eÅ^−3^

### DFT calculations

The interaction energies were evaluated at the RI-PB86-D3/def2-TZVP^34–36^ level of theory by means of the Turbomole 7.0 program^37^ and using the crystallographic coordinates. The D3 dispersion^36^ correction scheme was applied to the interaction energies because it is convenient for the correct evaluation of noncovalent interactions, specially π-stacking interactions. For the Mn(ii) metal centre (compound 1) the high spin configuration was used. The QTAIM^38^ analyses were used to characterize the NCIs using the RI-PB86-D3/def2-TZVP wavefunction.

The independent gradient model plot (IGM Plot^39^), an improved NCIPlot tool, has been used to identify and quantify the noncovalent interactions. The IGM-δg approach is based on a new definition of a non-interacting system in terms of electron density contra-gradience (electron density clash between two sources). A new descriptor, δg, locally accounts for the difference between a virtual upper limit of the electron density gradient (∇*ρ*IGM), representing a non-interacting system, and the true electron density gradient (∇*ρ*). Therefore, it provides an uncoupling scheme that automatically extracts the signature of interactions between two given fragments (δg_inter_), inside these fragments (δg_intra_), or even between two atoms (δg_pair_). The (δg_inter_) description has been used in this work, since we are interested in the intermoelcular interactions. Both the QTAIM and IGM-δg_inter_ analyses have been performed using the Multiwfn program^40^ and represented using the VMD visualization software.^41^

### Biological studies

#### Cell culture methods

Human breast cancer cells MDA-MD-231 (ATCC HTB-26), human breast cancer cells MCF-7 (ATCC HTB-22), human cervix epithelial carcinoma HeLa (ATCC CCL-2), human colon cancer cell line HT-29 (ATCC HTB-38), mouse neuroblastoma cell line Neuro-2a (ATCC CCL-131) and mouse fibroblast L-929 cell line (ATCC CCL-1) were obtained from the American Type Culture Collection (ATCC; Manassas, VA, USA) and cultured at 37 °C in a humidified atmosphere of 5% CO_2_ in air. HeLa cells were cultured in Dulbecco's Modified Eagle's Medium (DMEM) with 0.1 mM nonessential amino acids, 2 mM l-glutamine, 1.0 mM sodium pyruvate and 5% fetal bovine serum, at 37 °C in an atmosphere of 5% CO_2_. Cells were plated in 96-well sterile plates at a density of 1 × 10^4^ cells per well in 100 μL of medium and incubated for 24 h. Also MDA-MD-231, HT-29, MCF-7 and Neuro-2a were cultured in DMEM containing 10% fetal bovine serum, 100 units per ml of penicillin and 100 μg ml^−1^ of streptomycin. L-929 cells were cultured in RPMI-1640 medium containing 10% fetal bovine serum, 100 units per ml of penicillin and 100 μg ml^−1^ of streptomycin.

#### MTT assay in human cancer cell lines

Complexes 1–3 ([Mn_2_(pydco)_2_(bpy)_2_(H_2_O)_2_]·2H_2_O (1), [Zn(bpy)(Hpydco)_2_] (2) and [Zn(bpy)Cl(Hpydco)]·2H_2_O (3)) were screened for antiproliferative activity against human breast cancer cells (MDA-MD-231), human cervix epithelial carcinoma (HeLa), human colon cancer cell line (HT-29), human breast cancer cells (MCF-7), mouse neuroblastoma cell line (Neuro-2a) and mouse fibroblast L-929 cell line using cisplatin as a comparative standard. Cell viability was evaluated by a colorimetric method based on the tetrazolium salt MTT ([3-(4,5-dimethylthiazol-2-yl)-2,5-diphenyltetrazolium bromide]), which is reduced by living cells to yield purple formazan crystals. Cells were seeded in 96-well plates at a density of 2–5 × 10^4^ cells of MDA-MD-231, MCF-7, HeLa, HT-29, Neuro-2a and L-929 per well in 200 μL of culture medium and left to incubate overnight for optimal adherence. After careful removal of the medium, 200 μL of a dilution series of the complexes in fresh medium were added and incubation was performed at 37 °C/5% CO_2_ for 72 h. Complexes 1–3 were first solubilized in DMSO, diluted in medium and added to the cells in final concentrations between 20 nM and 200 μM. The percentage of DMSO in the cell culture medium did not exceed 0.5%. Cisplatin was first solubilized in saline and then added at the same concentrations used for the other complexes. At the end of the incubation period, the complexes were removed, and the cells were incubated with 200 μL of MTT solution (500 μg ml^−1^). After 3–4 h at 37 °C/5% CO_2_, the medium was removed, and the purple formazan crystals were dissolved in 200 μL of DMSO by shaking. The cell viability was evaluated by measurement of the absorbance at 570 nm by using a STAT FAX-2100 microplate reader (Awareness Technology, Palm City, FL, USA). The cell viability was calculated by dividing the absorbance of each well by that of the control wells (cells treated with medium containing 1% DMSO). Each experiment was repeated at least three times and each point was determined in at least three replicates.

#### Apoptosis assay for 2 and 3 by flow cytometry

In order to study in which way 1–3 produced the cellular death (necrosis or apoptosis) studies of flow cytometry were performed on the complexes and cisplatin as a reference. These complexes were incubated for 24 h at a concentration close to the IC_50_ and the results are shown in Fig. 5 and 6. Four areas in the diagrams stand for necrotic cells (Q1, low Annexin V-FITC and high PI signal, left square on the top), late apoptosis or necrosis cells (Q2, high Annexin V-FITC and high PI signal, right square on the top), live cells (Q3, low Annexin V-FITC and low PI signal, left square at the bottom), apoptosis cells (Q4, high Annexin V-FITC and low PI signal, right square at the bottom), respectively. As shown in Fig. 5 and 6 and Tables 4 and 5, 2 and 3 could induce apoptosis against MDA-MB-231 cancer cell lines but the proapoptotic property needs further investigation to better understand the precise mechanism of action of the complexes.

**Analysis of apoptosis for 2 and 3 by cytometry using TUNEL assay.** Also, apoptosis was detected using an *in situ* cell death detection kit (Boehringer Mannheim Corp., Indianapolis, IN) as described by Narla *et al.* and Zhu *et al.* (Narla *et al.*, 2000;^42^ Zhu *et al.*, 1998 (ref. 43)). Cells were incubated with 1–3 in 0.3% DMSO or 1 : 16-diluted plasma samples from DFX-treated mice for 48 h at 37 °C and were fixed, permeabilized and incubated with the reaction mixture containing TdT- and FITC-conjugated dUTP, and counterstained with propidium iodide. Cells were transferred to slides and viewed with a confocal laser scanning microscope (Bio-Rad MRC 1024) mounted on a Nikon Eclipse E800 series upright microscope as reported previously (Narla *et al.*, 2000;^42^ Zhu *et al.*, 1998 (ref. 43)).

#### Statistical analysis

IC_50_ values are expressed as mean ± standard deviation (SD) from at least three independent experiments. Statistical tests including One way ANOVA, Tukey multiple comparison or unpaired Student's *t*-tests were performed using SPSS, ver.17 software. A *p* value of less than 0.05 was considered as significant.

## Results and discussion

### Synthesis

Complexes 1–3 were synthesized by the reaction of Zn and Mn salts, H_2_pydco, and bpy at elevated temperature (see the Experimental section for details). Careful control of reaction time and solvent type are key factors to obtain the complexes reported herein. It is important to note that by changing the accompanied anion from NO_3_^−^ to Cl^−^, 3 was obtained. The crystallization time of 1–3 was a few days by slow evaporation of mother liquor. Also, powder X-ray diffraction further demonstrated the bulk purity of 1–3 (Fig. S1–S3† (in the ESI)).† Complexes 1–3 are soluble in water, DMSO and are stable in air.

### IR spectra

The infrared spectra of 1–3 are consistent with the structures determined by single-crystal X-ray diffraction (See Table 2 and Fig. S4†). The broad and strong bands at 3000–3500 cm^−1^ can be attributed to the O–H stretching vibrations of lattice and coordinated water molecules and the hydroxyl groups of Hpydco^−^. They are also indicative of the presence of hydrogen bonds.^12,44^ In comparison with free H_2_pydco (1725 and 1413 cm^−1^), the strong asymmetric and symmetric stretching vibrations of the COO^−^ group in 1–3 are shifted to the lower wavenumbers in the range of 1650–1640 cm^−1^ and 1400–1390 cm^−1^, respectively. The separation between *ν*_as_(COO^−^) and *ν*_s_(COO^−^) has been often used to diagnose the coordination modes for carboxylate ligands. The separation for monodentate carboxylate groups is >200 cm^−1^, whereas it is >200 cm^−1^ in bidentate ones. In our complexes this separation shows that the carboxylate group is coordinated monodentately to the metal centre in agreement with the crystal structure. Also, bands in the 1255–1207 cm^−1^ region were assigned to the N–O stretching vibrations of the pyridine-*N*-oxide.^45,46^

**Table 2:** Representation of important absorption bands (cm^−1^) for 1–3 and free H_2_pydco for comparison

Compound	*ν*(OH)	*ν*_as_(COO^−^)	*ν*_s_(COO^−^)	*ν*(N–O)
1	3481	1640	1390	1255
2	3130	1650	1395	1210
3	3487	1644	1400	1207
H_2_pydco	3138	1725	1413	1229

### Thermogravimetric study

The thermal stability of 1–3 has been studied by thermogravimetric analysis (TGA) under an air atmosphere. The TG curve of 1 exhibits two weight-loss events (Fig. S5†). The first stage occurs between 25 and 165 °C which corresponds to the removal of three uncoordinated and coordinated water molecules amounting to 6.30 (calcd 6.27%) and the second stage is a double stage sequential process from 170 to 450 °C, due to the combustion of the two pydco^2−^ and two bpy ligands with a weight loss of 79.50 (calcd 78.21%). For 2, the TG curve exhibits a one-step weight loss that is in consistent with its formula (without coordinated or uncoordinated water molecules), occurring between 25 and 540 °C which is attributed to the decomposition of the two Hpydco^−^ and one bpy ligands with a weight loss of 82.76 (calcd 89.57%). Unlike those for 1 and 2, the TG curve for 3 exhibits a three-step weight loss process. The weight losses of 7.58, 7.47, and 71.58%, occurring in the temperature ranges of 25–200, 200–221, and 220–450 °C, respectively, can be attributed to the removal of two uncoordinated water molecules, Cl^−^ as well as Hpydco^−^ and bpy ligands (calcd 7.21, 7.40 and 68.21%).

### Structure description of 1 and 2

Crystallographic details can be found in the CIF files. The CIF files are available free of charge from the Cambridge Crystallographic Data Centre CCDC^47^ (1934944, 1979889 and 2009484). Structural analysis indicates 1–3 contain discrete complexes containing *N*-donor bpy and *O*-donor pydco^2−^ (or Hpydco^−^) ligands. Selected bond distances and angles and a list of hydrogen bond geometries are reported in Tables S1 and S2.†

#### Crystal structure of 1

**Fig. 1:**
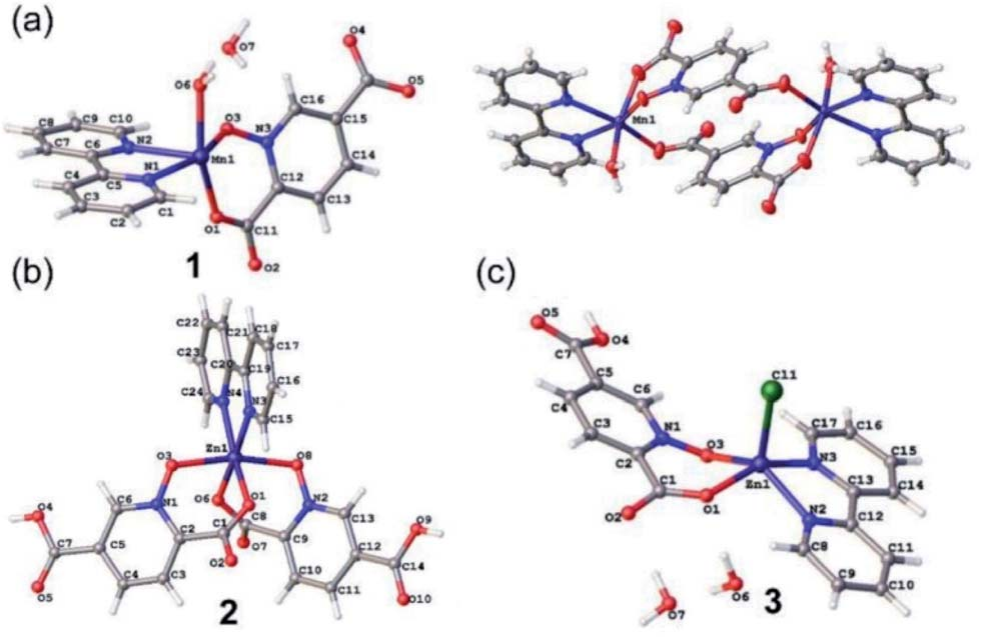
The asymmetric units of 1 (a), 2 (b) and 3 (c) with selected atoms labeled. The centrosymmetric dimer of 1 is also shown (top right).

**Fig. 2:**
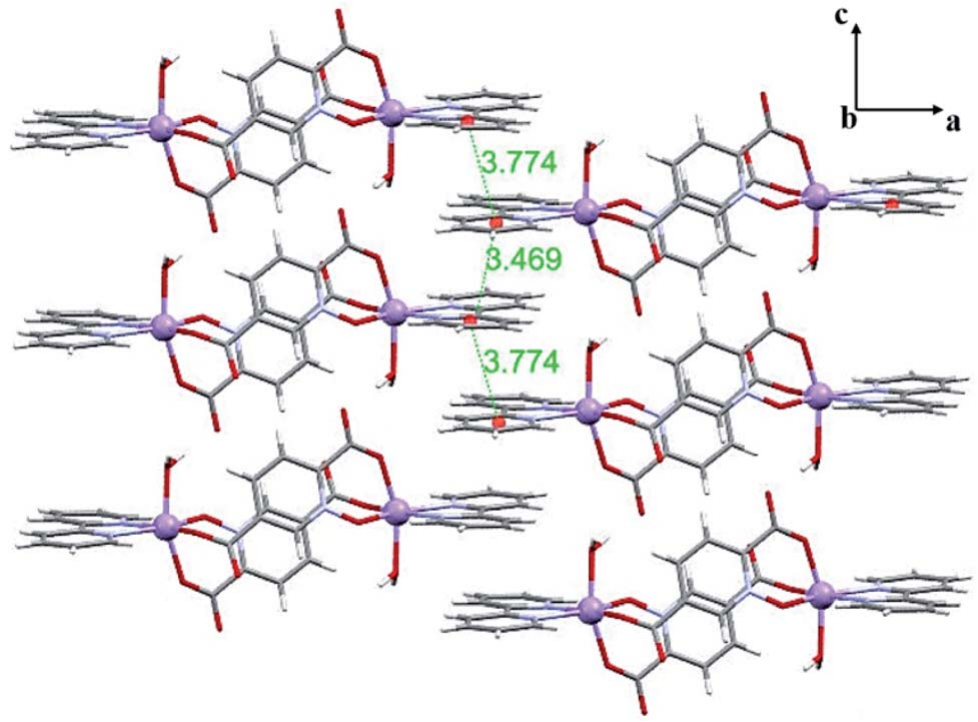
2D representation of 1 in the *ac* plane formed by π-interactions (distances are shown in Å).

Complex 1 has a dimeric structure [Mn_2_(pydco)_2_(bpy)_2_(H_2_O)_2_]·2H_2_O in which pydco^2−^ acts as a bridge between two monomers by its carboxylate group in the 5-position. The asymmetric unit of 1 is composed of a half of the molecule including one Mn(ii) ion, pydco^2−^ and bpy ligands as well as one coordinated and one uncoordinated water molecule (Fig. 1a). Each Mn centre displays a distorted octahedral coordination geometry (Table 3) in which the axial positions are occupied by O_water_ and O_carboxylate_, and the equatorial positions are occupied by N1 and N2 from a bpy, O_carboxylate_ from adjacent pydco^2−^, and O_*N*-oxide_ (Fig. 1). As shown in Fig. 2, π-interactions between aromatic rings are excellent structural-directing agents that link neighbouring dimers to each other to create 2D-supramolecular layers in the *ac* plane (*d*_centre_ = 3.77 Å and *d*_plain_ = 3.53 Å and the *α* angle is 23.07°). Furthermore, intermolecular hydrogen bonds can connect these layers to create a 3D-supramolecular architecture (O–H⋯O hydrogen bonds between the uncoordinated water molecules and coordinated water of one dimer and the 5-carboxylate oxygen atom from another dimer) (Fig. S6†).

**Table 3:** The IC_50_ ± SD (μM) values^a^ in various cell lines for the synthesized complexes

Compound	MCF-7	MDA-MD-231	HT-29	HeLa	Neuro-2a	L929
1	56.6 ± 7.78	28.9 ± 2.47	57.9 ± 4.71	39.2 ± 2.98	79.5 ± 8.44	>100
2	15.6 ± 3.24	5.94 ± 1.98	17.3 ± 2.57	41.2 ± 5.07	81.2 ± 9.28	>100
3	17.9 ± 2.66	9.18 ± 2.25	14.6 ± 3.55	37.6 ± 4.61	87.7 ± 6.93	>100
H_2_pydco	57.9 ± 5.33	45.7 ± 4.75	58.9 ± 5.01	67.1 ± 5.45	>100	>100
bpy	51.3 ± 4.78	24.9 ± 1.98	67.8 ± 5.54	46.5 ± 4.03	97.3 ± 8.70	>100
Cisplatin	5.94 ± 1.47	24.7 ± 4.71	19.3 ± 3.46	0.45 ± 0.13	103 ± 9.8	0.7 ± 0.2

a The concentration of the complex required to inhibit cell growth by 50%. The experiments were done in triplicate. Data were expressed as the mean of the triplicate. IC_50_ > 100 μM is considered to be inactive.

#### Crystal structure of 2

**Fig. 3:**
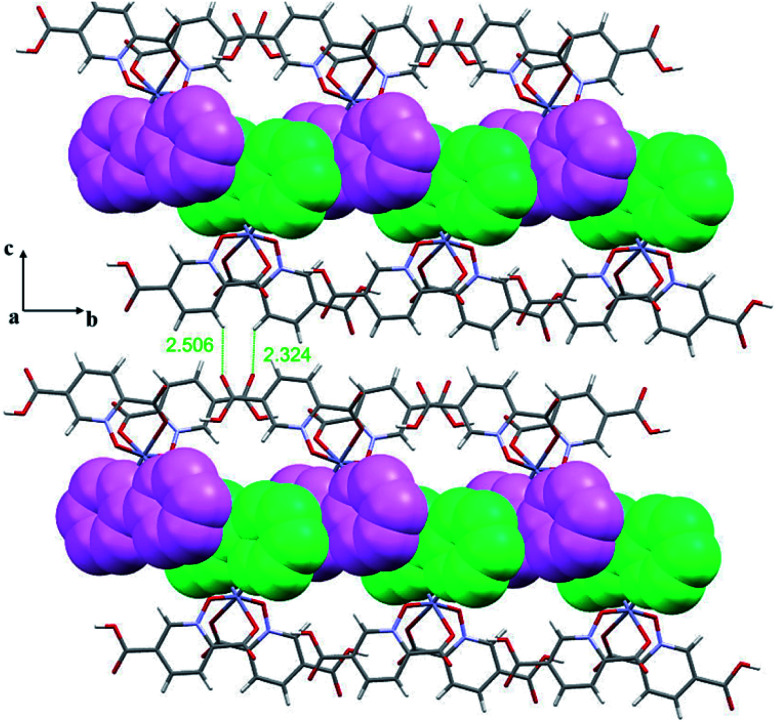
2D representation of 2 in the *bc* plane formed by π-interactions between adjacent bpy rings where bpy rings from up and down monomers are shown in different colours (purple and green) as well as intermolecular C–H⋯O hydrogen bonding (distances are shown in Å).

Complex 2 has a monomeric structure with the [Zn(bpy)(Hpydco)_2_] formula. The asymmetric unit of 2 is composed of one Zn(ii) ion, two bidentate Hpydco^−^ ligands and one bpy ligand (Fig. 1b). The zinc centre displays a distorted octahedral coordination geometry (Table 3) in which the axial positions are occupied by O3 and O8 from *N*-oxide groups of two Hpydco^−^ ligands, and the equatorial positions are occupied by N3 and N4 from the bpy ligand as well as O1 and O6 from carboxylate groups of two Hpydco^−^ ligands (Fig. 1). It is important to note that extensive π-interactions between adjacent bpy rings (*d*_centre_ = 3.502 Å and *d*_plain_ = 3.211 Å and the *α* angle is 24.5°) as well as intermolecular C–H⋯O hydrogen bonds can form 2D-supramolecular layers in the *bc* plane (Fig. 3).

#### Crystal structure of 3

**Fig. 4:**
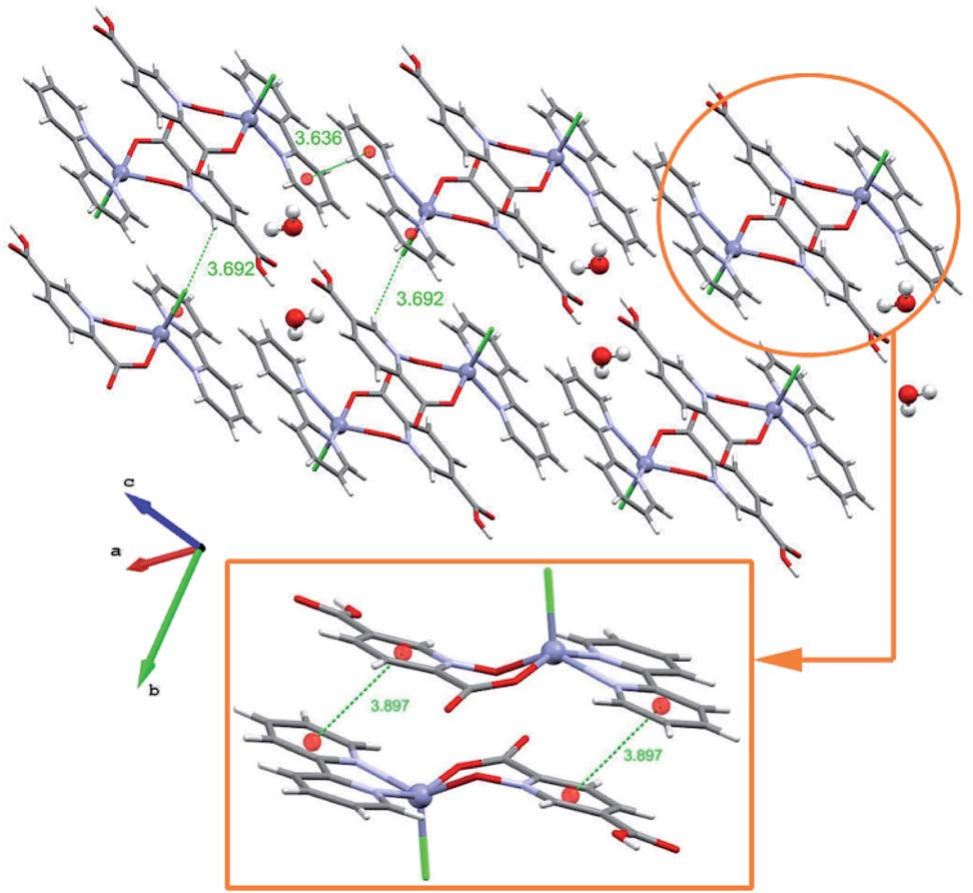
2D representation of 3 that is formed by extensive π-interactions as well as intermolecular O–H⋯O hydrogen bonds (distances are shown in Å).

Complex 3 has a monomeric structure with the [Zn(bpy)Cl(Hpydco)]·2H_2_O formula. The asymmetric unit of 3 is composed of one Zn(ii) ion, a bidentate Hpydco^−^ ligand, one bpy ligand, one chloride anion and two uncoordinated water molecules (Fig. 1c). The zinc centre displays a distorted square pyramidal coordination geometry (Table 3) in which the axial position is occupied by a chloride and the equatorial plane is occupied by two nitrogen atoms of bpy and two oxygen atoms of Hpydco^−^ (Fig. 1). As depicted in Fig. 4 π-interactions (*d*_centre_ = 3.89, 3.63 Å and *d*_plain_ = 3.48, 3.63 Å and the *α* angle is 34.9°, 23.8°) between aromatic rings of monomeric units formed 1D chains. Furthermore, C–H⋯π interactions (3.692 Å) as well as strong O–H⋯O hydrogen bonds (1.669 Å) between uncoordinated water molecules and Hpydco^−^ lead to the formation of a 2D-supramolecular structure. Interestingly, the chloride anion and Hpydco^−^ ligand through uncoordinated water molecule (O–H⋯Cl, O–H⋯O and C–H⋯O hydrogen bonds) formed 1D-chains along the *b* axis (Fig. S7†).

#### Structural comparison

(i) Auxiliary ligand: investigations revealed that *N*-bidentate ligands such as 1,10-phenanthroline (phen) and 2,2′-bipyridine show same behaviour, due to the formation of five-membered chelate rings (see Table S3†).^48^ It is important to note that by replacing bpy instead of phen^29^ a similar dimeric structure was obtained. (ii) Coordination modes of metallic centre and organic ligands: the presentation of auxiliary ligand (bpy) in all three complexes is similar (see Table S3†). However, the coordination mode of H_2_pydco for 1 is different from those in 2 and 3. H_2_pydco mostly acts as chelate ligand *via N*-oxide and the neighbouring carboxylate functional group, although it can form an adduct from the other carboxylate of the molecule in some cases. The formation of dimeric complex in 1 can be explained through this semi-bridge ligand. However, a similar framework was formed *via* π⋯π interactions in the packing of 3. In contrast to 1 and 2, complex 3 crystallized with a distorted square pyramidal geometry with coordination number of five and *τ*_5_ of 0.3053. This geometry is likely the result of the presence of the stronger ligand chloride in the molecular structure which would render a second potential H_2_pydco ligand monodentate and perhaps too weakly coordinated.

### DFT analysis of noncovalent interactions

In this section we have used the IGM plot method to identify the noncovalent interactions in real space. This representation uses a colour code to identify attractive and repulsive noncovalent contacts that is based on the sign of the middle eigenvalue of the Hessian of *ρ*(*λ*_2_). Blue and green are used to represent strong and weak attractive interactions, respectively and yellow and red to indicate weak and strong repulsive interactions, respectively.

The study is focused on the comparison of the relevant structure-guiding π-stacking interactions observed in compounds 1–3, as highlighted before in Fig. 2–4. For compound 1 the QTAIM analysis shows the existence of several bond CPs (small red spheres), bond paths and an extended green IGM isosurface connecting the pypiridine ligands. Moreover, two symmetrically equivalent CH⋯O HBs are also disclosed, each one characterized by a bond CP, bond path and small green isoosurface interconnecting the O and H-atoms. We have evaluated the dissociation energy of each H-bond by using the equation proposed by Espinosa *et al.* (*E*_dis_ = ½ × Vr^49^) that uses the potential energy density (*V*_r_) at the bond CP that characterizes the HB. As a result, the energy of the CH⋯O HBs in 1 is 0.9 kcal mol^−1^ (1.8 kcal mol^−1^ the total HB contribution), thus evidencing that the formation of the dimer is dominated by the π-stacking interaction. Such strong interaction energy of 1 (Δ*E*_1_ = −32.9 kcal mol^−1^) is due to the antiparallel orientation of the molecules that maximizes the dipole···dipole interaction. In this type of complexes, where the aromatic rings are coordinated to metal centres, the π-system is very polarized, generating large dipole···dipole attractions, as previously demonstrated.^50–54^ In compound 2, a similar combination of interactions is observed. That is, the QTAIM/IGM plot analysis shows the existence of a π-stacking interaction characterized by two bond CPs and bond paths connecting two atoms of each aromatic ring and two CH⋯O interactions characterized by the corresponding bond CPs and bond paths. All interactions are also confirmed by the IGM plot isosurfaces. In this case one H-bond is stronger (blue isosurface) with a concomitant dissociation energy of 2.1 kcal mol^−1^. The other H-bond is weaker (0.7 kcal mol^−1^) in line with the green colour and small size of the IGM surface. In this case the interaction energy is moderately strong (Δ*E*_2_ = −18.0 kcal mol^−1^) because the overlap is of the π-system is smaller compared to 1.

**Fig. 5:**
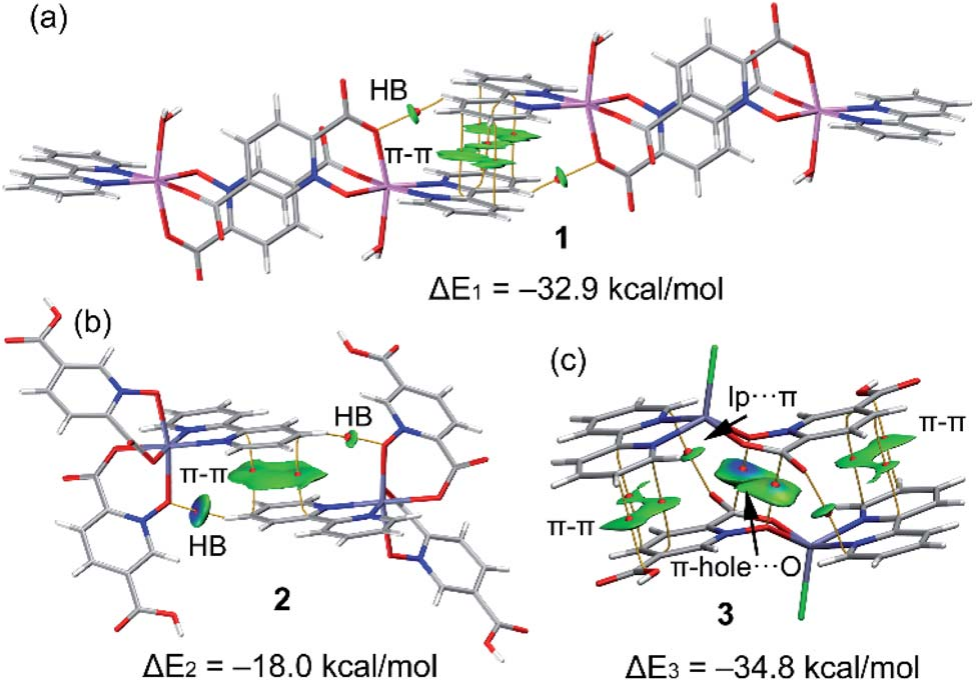
Combined QTIAM (bond CPs in red and bond path as orange lines) and IGM plot (IGM-δg_inter_ isosurface 0.085) and colour range −0.03 a. u. ≤ (sign*λ*_2_)*ρ* ≤ 0.03 a. u. The dimerization energies are also indicated. Level of theory: PBE0-D3/def2-TZVP.

Finally, in compound 3, the π-stacked dimer represented in Fig. 5c shows an intricate combination of interactions that explains the strong interaction energy (Δ*E*_3_ = −34.8 kcal mol^−1^). That is, in addition to the π-stacking interactions between the π-clouds of the bipyridine and pyridine-2,5-dicarboxylic acid *N*-oxide ligands, the QTAIM analysis shows the existence of two remarkable π-hole⋯O interactions between the O-atoms of the *N*-oxide and the carbonyl C-atoms of the coordinated carboxylate groups, characterized by the corresponding bond CPs and bond paths interconnecting the O and C-atoms. Moreover, the IGM plot reveals a blue isosurface between the C and O-atoms confirming the existence and strong nature of these contacts. Furthermore, the QTAIM distribution of bond CPs reveals also the existence of a lp⋯π interaction between the non-coordinated O-atom of the carboxylate group and the π-system of the bipyridine ring.

### Biological studies

Clearly from Table 3, all three complexes exhibited remarkable cytotoxic activity against cancer cell lines. In some cases, the IC_50_ values were lower than those of cisplatin indicating better antiproliferative effects. Among the complexes, 2 and 3 displayed the best results in terms of cytotoxicity. It seems that the presence of the bioactive metal, zinc, H_2_pydco and bpy ligands play a decisive role in generating cytotoxic effects and consequently antiproliferative activities.

Cytotoxic activity was simultaneously measured for mouse fibroblast normal cell line (L929) as control. As shown in Table 4, 2 and 3, displayed cytotoxic activities against L929 significantly higher than against cancer cell lines making them appropriate candidates for anticancer drugs. A notable point is that in the case of cisplatin, the IC_50_ value against normal cell L929 was so low that it was unable to make a distinction between normal and cancer cells.

**Table 4:** Percentages of the cell death pathways observed by the flow cytometry assay for 2

Treatment	Vital cells (%)	Apoptotic cells (%)	Late apoptotic/necrotic cells (%)	Necrotic cells (%)
Control	81.13	8.68	9.45	0.74
Cisplatin	33.74	34.65	30.63	0.98
2	19.73	43.91	35.54	0.82

In this study, the potential of three complexes of Mn and Zn as effective antiproliferative agents have been investigated *in vitro*. In order to assess this, the *in vitro* cytotoxicity of these complexes, against MDA-MDA-MD-231, MCF-7, HeLa, HT-29, Neuro-2a and L-929 cell lines were determined by MTT-based assays (Table 3). For 1–3, and cisplatin as a comparative standard, we have measured IC_50_ values for all cell lines. The measurements were done after 72 h of incubation using concentrations of the several complexes in the range 20 nM and 200 μM. The values determined for these complexes spanned between 5.94 to 100 μM for 2 and 3, while those found for cisplatin as a comparative standard ranged between 0.7 to 100 μM (Table 3).

Based on the results of the *in vitro* cytotoxicity studies of 2 and 3, those systems having maximum cytotoxicity were selected for apoptosis assay by flow cytometry.

**Fig. 6:**
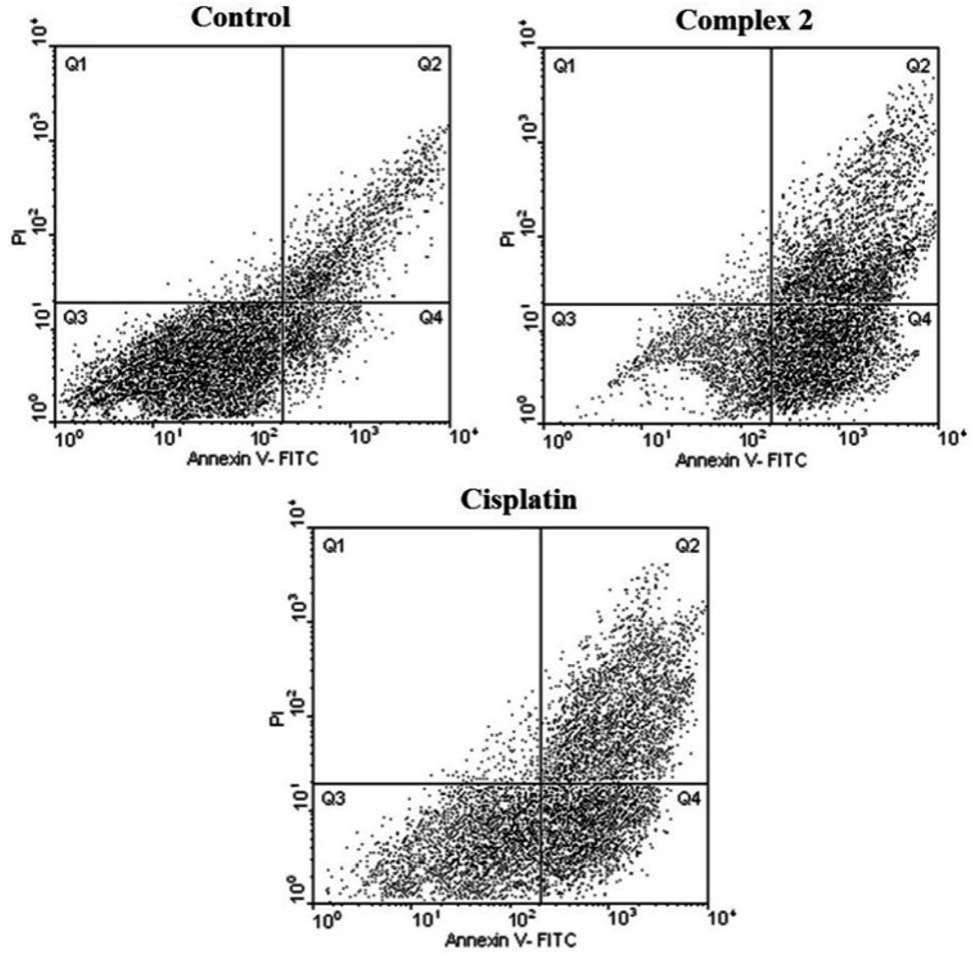
Flow cytometric results after the exposure of MDA-MD-231 cancer cells to 2 and to cisplatin. Four areas in the diagrams represent four different cell states: necrotic cells (Q1), late apoptotic or necrotic cells (Q2), living cells (Q3) and apoptotic cells (Q4).

**Fig. 7:**
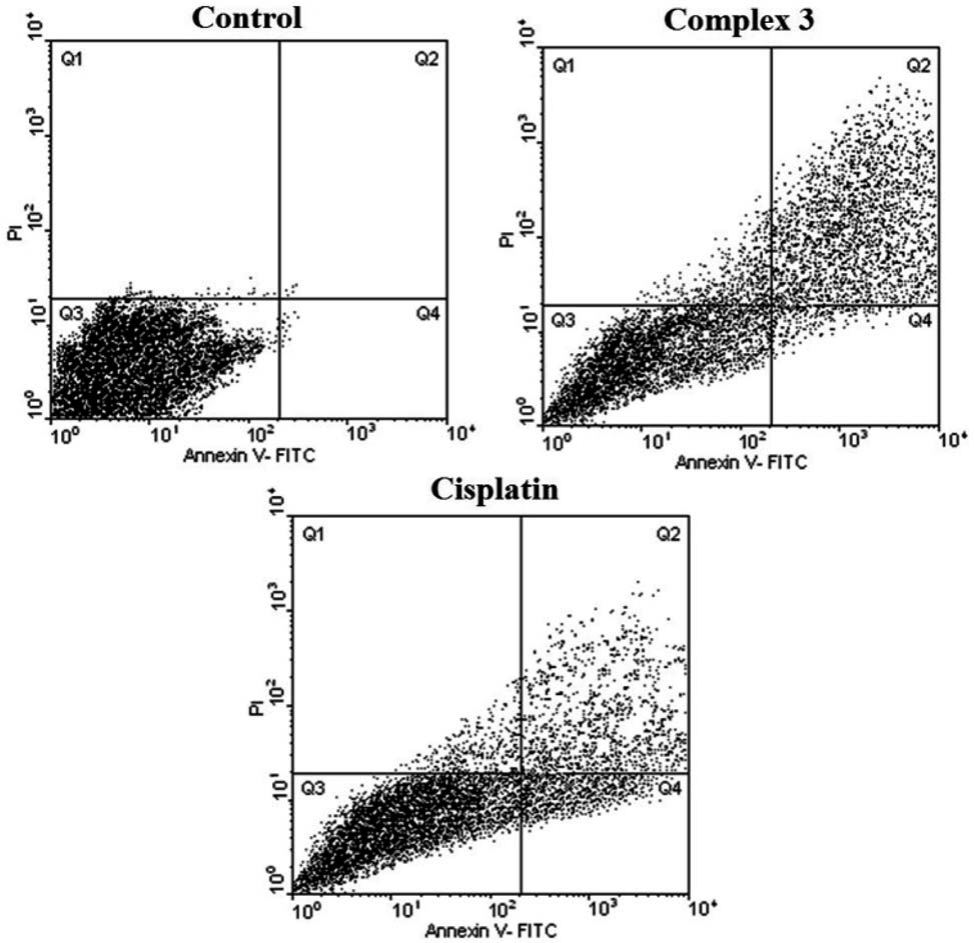
Flow cytometric results after the exposure of MDA-MD-231 cancer cells to 3 and cisplatin. Four areas in the diagrams represent four different cell states: necrotic cells (Q1), late apoptotic or necrotic cells (Q2), living cells (Q3) and apoptotic cells (Q4).

As seen in Tables 4, 5, Fig. 6 and 7, 2 showed a high population of apoptotic cell (79.45%), nearly 1.2-fold higher than cisplatin (65.28%) at the same concentration. Also according to Table 5 and 3 showed a high population of apoptotic cell (63.3%), nearly 1.8-fold higher than cisplatin (35.8%) at the same concentration against MDA-MB-231 cancer cell line.

**Table 5:** Percentages of the cell death pathways observed by the flow cytometry assay for 3

Treatment	Vital cells (%)	Apoptotic cells (%)	Late apoptotic/necrotic cells (%)	Necrotic cells (%)
Control	99.00	0.30	0.10	0.60
Cisplatin	61.00	15.80	20.00	3.20
3	32.00	4.30	59.00	4.70

**Fig. 8:**
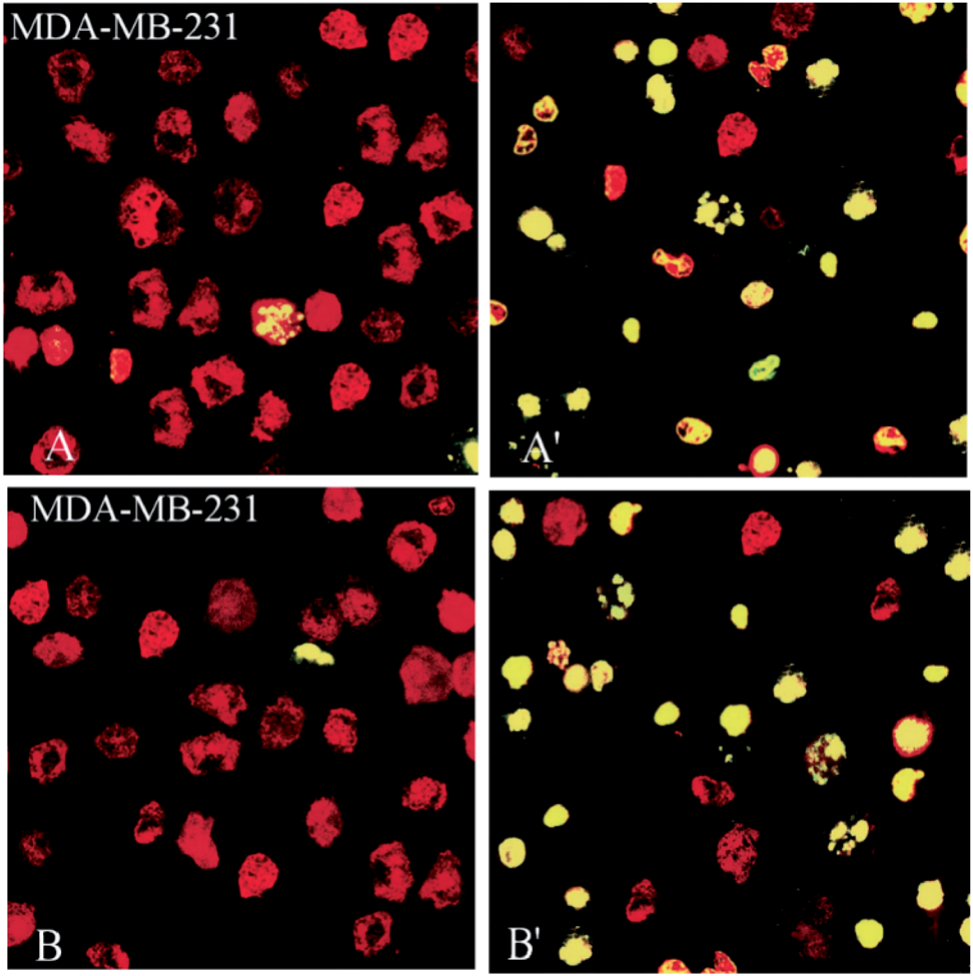
Compounds 2 and 3, induce apoptosis in human breast (MDA-MD-231) cancer cell line. MDA-MB-231 breast cancer (A, A′ for 2 and B, B′ for 3) cells were incubated with 10 mM of 2, 3 for 24 h, fixed, permeabilized and visualized for DNA degradation in a TUNEL assay using dUTP-labeling. Red fluorescence, nuclei stained with propidium iodide. Green or yellow (*i.e.*, superimposed red and green) fluorescence, apoptotic nuclei containing fragmented DNA. When compared with controls, treated with 0.3% DMSO (A and B), several of the cells incubated with 2 and 3 (A′, B′) exhibited apoptotic nuclei.

Also, 2- and 3-induced cell death was confirmed to be apoptotic using the TUNEL of exposed 3′-OH termini of DNA with dUTP-FITC. As shown in the confocal laser scanning microscopy images in Fig. 8, complex-treated MDA-MB-231 breast cancer cells, examined for dUTP-FITC incorporation (green fluorescence) and propidium iodide counterstaining (red fluorescence), exhibited many apoptotic yellow nuclei (superimposed green and red fluorescence) at 24 h after treatment.

The results demonstrated that 2 and 3 could induce apoptosis against MDA-MB-231 cancer cell line but the proapoptotic property needs further investigation to better understand the precise mechanism of action of these complexes and basic pre-clinical research is needed before they could be recommended for human administration.

## Conclusion

In this work, we report an approach where the combination of N- and O-donor ligands leads to interesting and diverse frameworks incorporating bio-essential metal ions such as manganese and zinc. This study demonstrates that supramolecular frameworks of 1–3 constructed by self-assembling through electrostatic interactions such as strong hydrogen bonds and a variety of π–π stacking interactions. Which have been further studied using DFT calculations including the IGM plot analysis. They revealed the existence of strong π-stacking interactions in the solid state of the three complexes and remarkable π-hole⋯O contacts between the *N*-oxide and carboxylate groups in compound 3. We have considered the antiproliferative effects of these complexes and compare their biological activity with each other. The obtained results showed that the effect of antiproliferative of 1–3 is due to the presence of metal centres and is somewhat influenced by the auxiliary ligand, and apparently the main ligand did not show any antiproliferative activity. Heteroleptic complexes are preferable to homoleptic complexes due to their size variation, type of interactions, effect on metal oxidation numbers, and many other factors.

## Author contributions

Hanie Alizadeh: methodology, formal analysis, investigation, data curation, writing-original draft preparation. Masoud Mirzaei: conceptualization, funding acquisition, main idea, supervision, writing-review and editing, project administration, visualization. Amir Sh. Saljooghi: biological studies, funding acquisition, supervision, writing-review and editing. Vida Jodaian: formal analysis, data curation. Maryam Bazargan: formal analysis, software, data curation, writing-original draft preparation. Joel T. Mague: crystallographer. Rosa M. Gomila: DFT studies. Antonio Frontera: DFT studies, funding acquisition, supervision, writing-review-review and editing, project administration, visualization.

## Conflicts of interest

There are no conflicts to declare.

## Supplementary Material

RA-011-D1RA08258B-s001

RA-011-D1RA08258B-s002
