# Supplementary material for: Coordination complexes of zinc and manganese based on pyridine-2,5-dicarboxylic acid N-oxide: DFT studies and antiproliferative activities consideration
Source: RSC Adv. 2021 Nov 22;11(59):37403–12. doi: 10.1039/d1ra08258b (PMC9043818; doi:10.1039/d1ra08258b)
Supplement: RA-011-D1RA08258B-s001 [file RA-011-D1RA08258B-s001.pdf]

Electronic Supplementary Material (ESI) for RSC Advances.
This journal is © The Royal Society of Chemistry 2021

Electronic Supporting Material

Coordination complexes of zinc and manganese based on pyridine-2,5-dicarboxylic acid *N*-oxide: DFT studies and antiproliferative activities consideration

Hanie Alizadeh[a], Masoud Mirzaei[a,*], Amir Sh. Saljooghi[a], Vida Jodaian[b], Maryam Bazargan,[a] Joel T. Mague,[c] Rosa M. Gomila[d] and Antonio Frontera[d]

[a]*Department of Chemistry, Faculty of Science, Ferdowsi University of Mashhad, Mashhad 9177948974, Iran*
E-mail: mirzaeesh@um.ac.ir

[b]*Department of Chemistry, Islamshahr Branch, Islamic Azad University, Islamshahr, Iran*

[c]*Department of Chemistry, Tulane University, New Orleans, LA, 70118, USA*

[d]*Departament de Química, Universitat de les Illes Balears, Crta de Valldemossa km 7.5, 07122 Palma de Mallorca (Baleares), SPAIN.*

Contents

Fig. S1. XRD pattern of **1** (simulated and experimental) 2

Fig. S2. XRD pattern of **2** (simulated and experimental) 2

Fig. S3. XRD pattern of **3** (simulated and experimental) 2

Fig. S4. FTIR spectra of H_2pydco, **1**, **2** and **3**....................3

Fig. S5. TGA curves of **1**, **2** and **3**....................3

Fig. S6. Representation of the hydrogen bonding interactions between the 2D supramolecular layers....................4

Fig. S7. Representation of the 1D linear chain along the *b* axis in **3**....................4

Table S1. Selected bond lengths (Å) and angles (°) for **1-3**....................5

Table S2. Selected Hydrogen bond geometry, lengths (Å) and angles (°) for **1-3**....................6

Table S3. Coordination modes of the metallic centres and organic ligands in **1-3**....................7

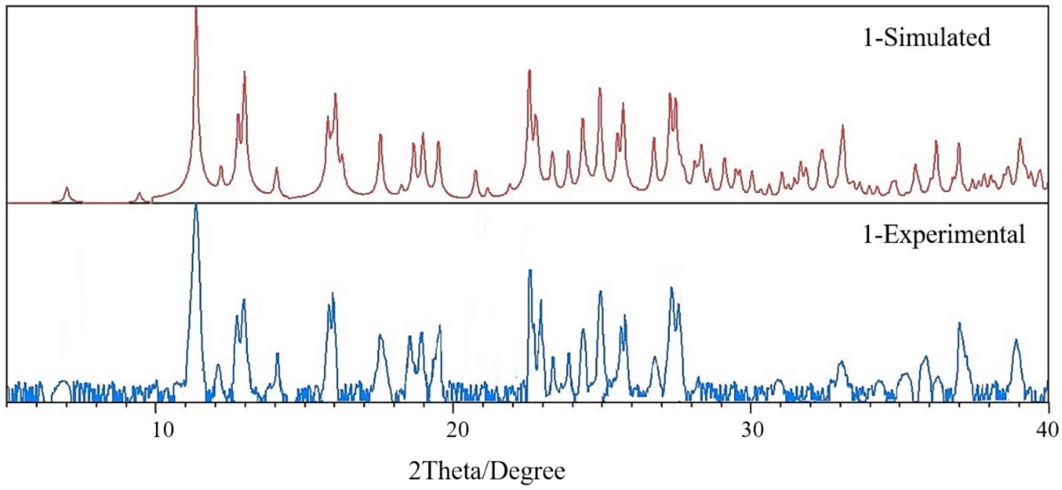

Fig. S1. XRD pattern of **1** (simulated and experimental).

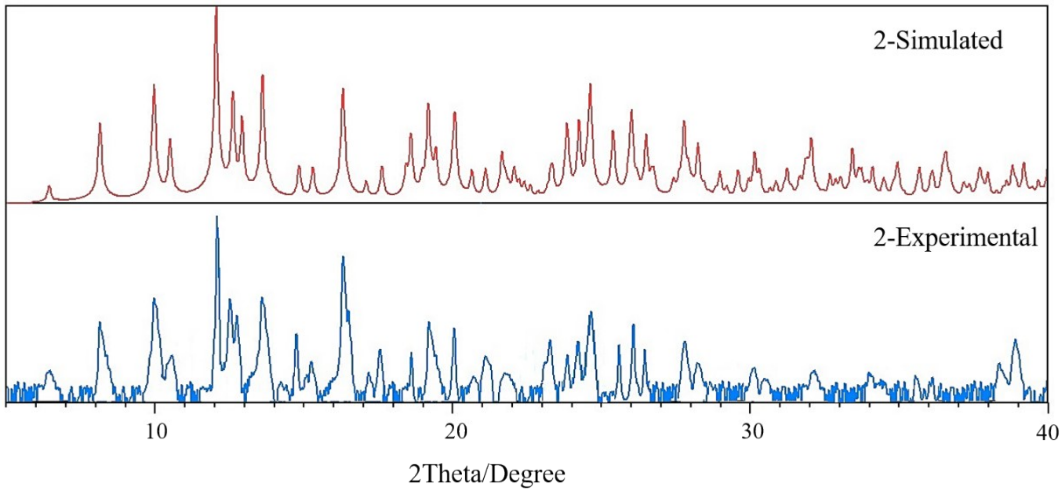

Fig. S2. XRD pattern of **2** (simulated and experimental).

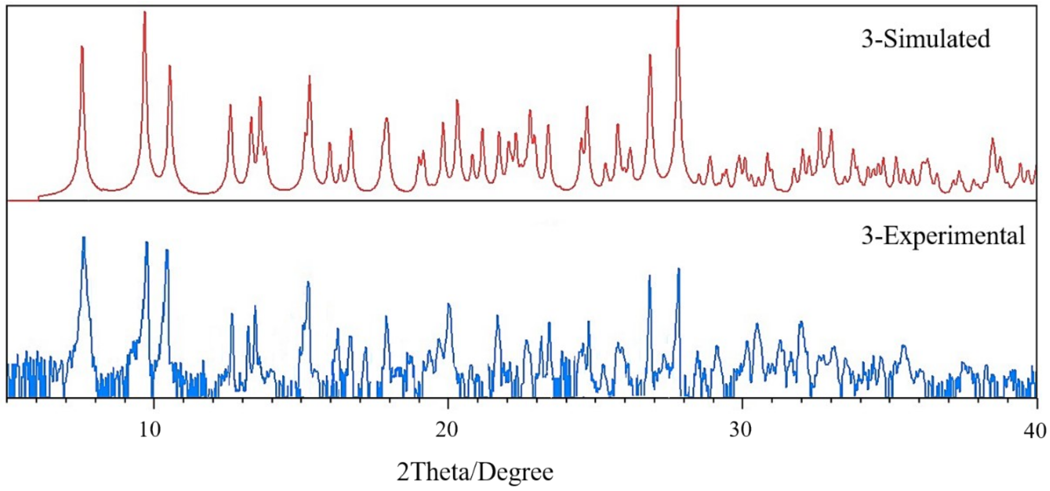

Fig. S3. XRD pattern of **3** (simulated and experimental).

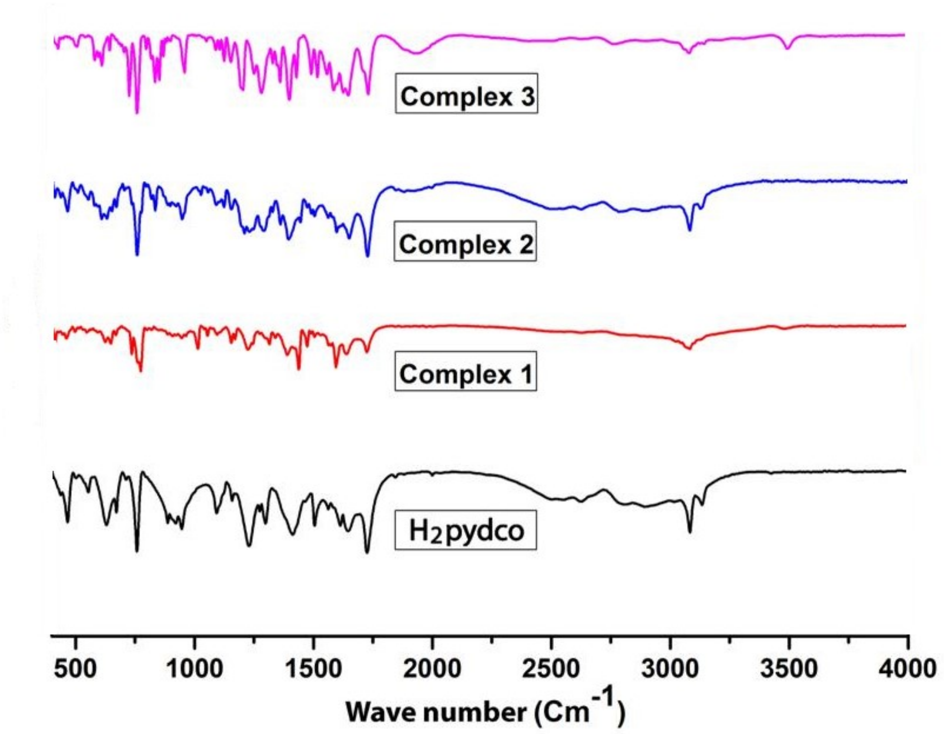

Fig. S4. FTIR spectra of H_2pydco, **1**, **2** and **3**.

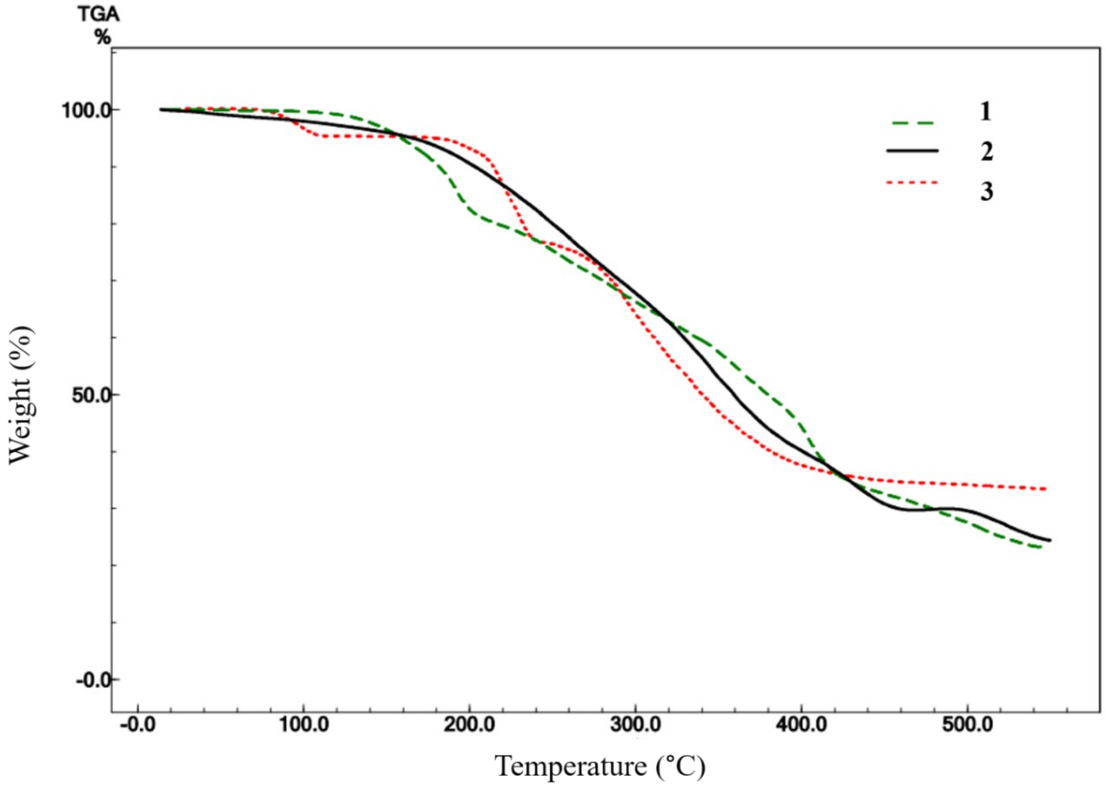

Fig. S5. TGA curves of **1**, **2** and **3**.

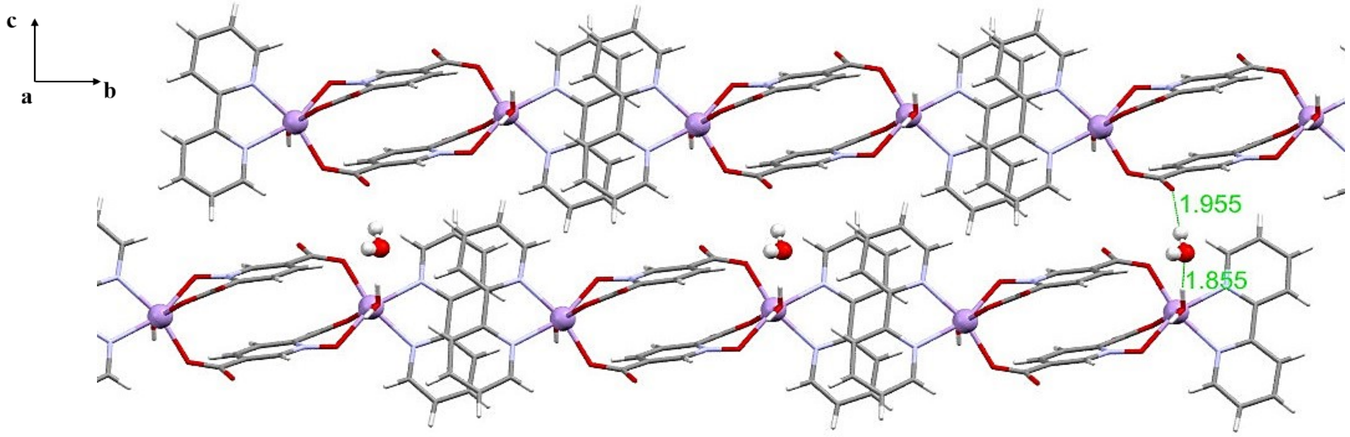
c
a
b
1.955
1.855

.

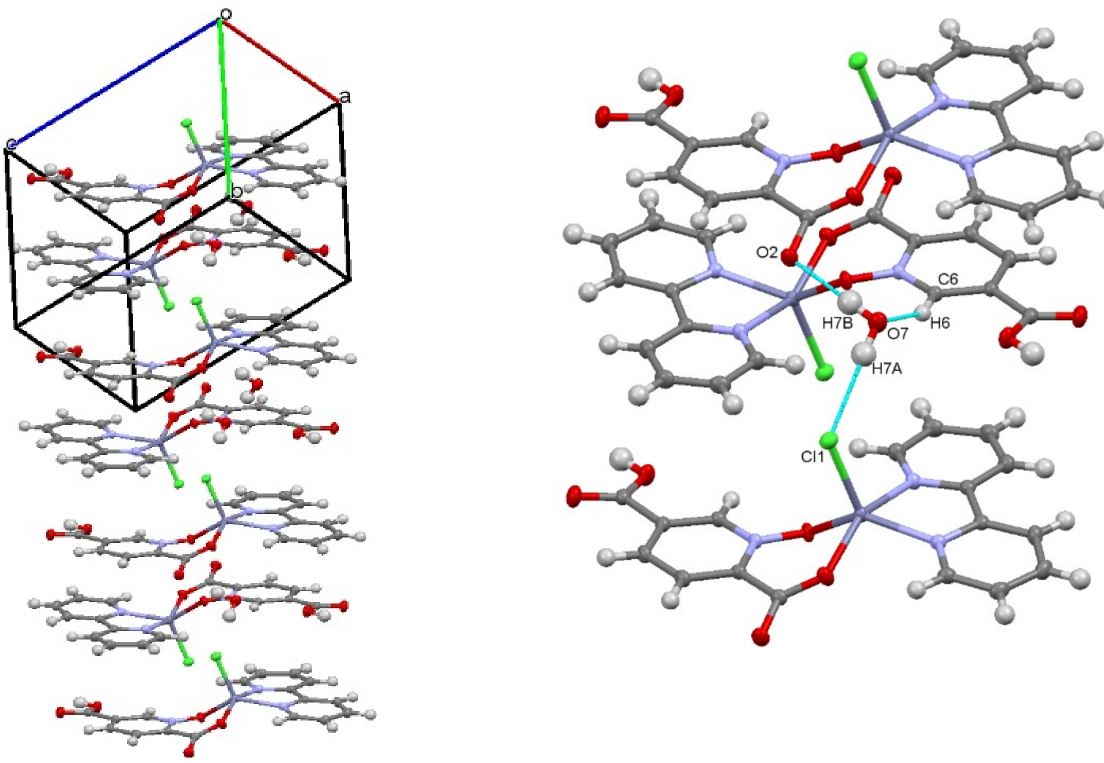

Fig. S7. Representation of the 1D linear chain along the *b* axis in **3**.

Table S1. Selected bond lengths (Å) and angles (°) for **1-3**.

**1**			
Mn1–O3	2.147(3)	O5–Mn1–O6	92.51(11)
Mn1–O1	2.126(3)	O5–Mn1–N1	91.37(11)
Mn1–O6	2.160(3)	O5–Mn1–N2	163.70(11)
Mn1–O5	2.141(3)	O1–Mn1–N2	95.52(12)
Mn1–N1	2.250(3)	N1–Mn1–N2	72.34(12)
Mn1–N2	2.272(3)	O1–Mn1–O3	78.26(11)
O1–Mn1–O5	89.12(12)	O3–Mn1–N1	159.19(12)
O1–Mn1–N1	104.64(11)	O1–Mn1–O6	159.05(11)
O3–Mn1–N2	86.90(11)	O6–Mn1–N2	88.73(11)
O5–Mn1–O3	109.36(11)	O6–Mn1–N1	96.21(11)
O3–Mn1–O6	81.51(10)	O5–Mn1–O6	92.51(11)
**2**			
Zn1–O1	2.0706(18)	N4–Zn1–O6	97.09(8)
Zn1–N3	2.101(2)	O1–Zn1–O3	82.63(7)
Zn1–N4	2.108(2)	N3–Zn1–O3	91.56(8)
Zn1–O6	2.1152(19)	N4–Zn1–O3	97.33(8)
Zn1–O3	2.120(2)	O6–Zn1–O3	88.81(7)
Zn1–O8	2.129(2)	O1–Zn1–O8	88.58(7)
O1–Zn1–N3	90.99(8)	N3–Zn1–O8	97.88(8)
O1–Zn1–N4	169.20(8)	N4–Zn1–O8	93.04(8)
N3–Zn1–N4	78.21(9)	O6–Zn1–O8	82.50(7)
O1–Zn1–O6	93.71(7)	O3–Zn1–O8	167.22(7)
N3–Zn1–O6	175.29(8)	N4–Zn1–O6	97.09(8)
**3**			
Zn1–O1	2.0242(10)	O3–Zn1–N3	87.54(4)
Zn1–N2	2.0951(12)	O1–Zn1–Cl1	101.86(3)
Zn1–Cl1	2.2739(4)	N2–Zn1–Cl1	115.44(3)
Zn1–O3	2.0251(10)	O1–Zn1–N2	89.51(4)
Zn1–N3	2.1186(12)	O1–Zn1–N3	153.79(5)
O1–Zn1–O3	86.15(4)	N2–Zn1–N3	77.38(5)
O3–Zn1–N2	135.47(4)	O3–Zn1–Cl1	108.81(3)

Complex	Coordination mode of metallic centre	Coordination mode of the main ligand	Coordination mode of auxiliary ligand
**1**	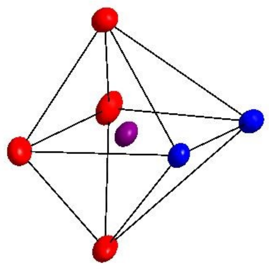	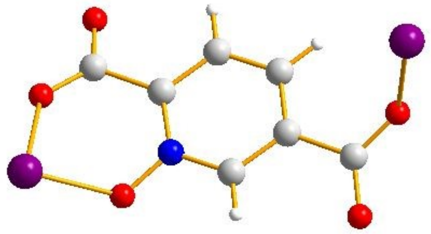	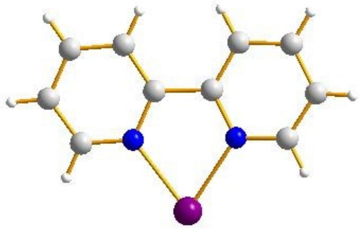

Table S2. Selected Hydrogen bond geometry, lengths (Å) and angles (°) for **1-3**.

D–H···A	D–H	H···A	D···A	D–H···A
**1**				
O6–H6A···O7	0.87	1.85	2.714(4)	168.8
O6–H6B···O2	0.87	1.87	2.742(4)	175.9
C4–H4···O6	0.95	2.55	3.399(5)	148.4
C7–H7···O7	0.95	2.55	3.269(5)	132.4
C10–H10···O3	0.95	2.50	3.102(5)	121.3
C16–H16···O2	0.95	2.56	3.377(5)	143.9
O7–H7A···O4	0.87	1.95	2.824(4)	177.3
O7–H7B···O4	0.87	2.04	2.870(4)	160.2
**2**				
O4–H4A···O2	0.87	1.69	2.556(3)	176.5
O9–H9···O6	0.87	1.77	2.631(3)	172.1
C3–H3···O5	0.95	2.32	3.084(3)	136.7
C10–H10···O10	0.95	2.51	3.330(3)	145.2
C23–H23···O7	0.95	2.42	3.283(4)	150.9
**3**				
O4-H4A···O6	0.87	1.67	2.5239(16)	167.0
C6-H6···O7	0.95	2.38	3.2645(18)	155.1
C11-H11···O5	0.95	2.49	3.3508(18)	151.4
O6-H6A···O7	0.87	1.82	2.6886(17)	177.8
O6-H6B···O5	0.87	1.97	2.8283(16)	169.5
O7-H7A···Cl1	0.87	2.34	3.2103(12)	177.2
O7-H7B···O2	0.87	1.89	2.7552(16)	178.4

**2**	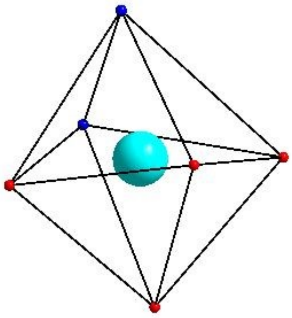	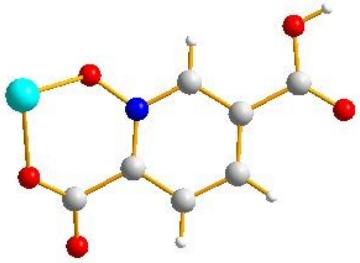	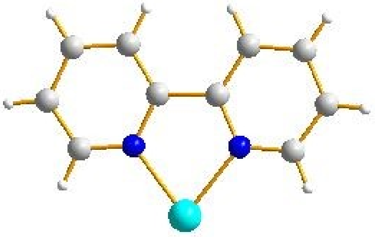
**3**	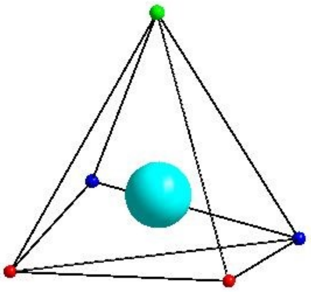	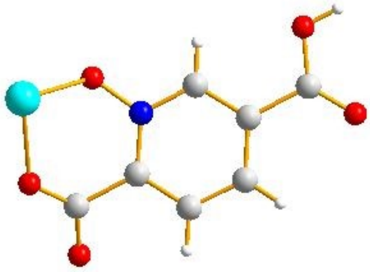	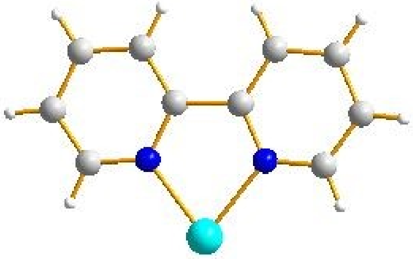

Table S3. Coordination modes of the metallic centres and organic ligands in **1-3**.
